# Impact of the Exposome on the Epigenome in Inflammatory Bowel Disease Patients and Animal Models

**DOI:** 10.3390/ijms23147611

**Published:** 2022-07-09

**Authors:** Sophie Vieujean, Bénédicte Caron, Vincent Haghnejad, Jean-Yves Jouzeau, Patrick Netter, Anne-Charlotte Heba, Ndeye Coumba Ndiaye, David Moulin, Guillermo Barreto, Silvio Danese, Laurent Peyrin-Biroulet

**Affiliations:** 1 Hepato-Gastroenterology and Digestive Oncology, University Hospital CHU of Liège, 4000 Liege, Belgium; s.vieujean@chuliege.be; 2 Department of Gastroenterology NGERE (INSERM U1256), Nancy University Hospital, University of Lorraine, Vandœuvre-lès-Nancy, F-54052 Nancy, France; caron.benedicte@hotmail.fr (B.C.); vincent.haghnejad@gmail.com (V.H.); 3 CNRS (French National Centre for Scientific Research), Laboratoire IMoPA, Université de Lorraine, UMR 7365, F-54000 Nancy, France; jean-yves.jouzeau@univ-lorraine.fr (J.-Y.J.); gotheilnetter@yahoo.fr (P.N.); david.moulin@univ-lorraine.fr (D.M.); guillermo.barreto@univ-lorraine.fr (G.B.); 4 NGERE (Nutrition-Genetics and Exposure to Environmental Risks), National Institute of Health and Medical Research, University of Lorraine, F-54000 Nancy, France; anne-charlotte.heba@univ-lorraine.fr (A.-C.H.); ndeye-coumba.ndiaye@univ-lorraine.fr (N.C.N.); 5 Lung Cancer Epigenetics, Max-Planck-Institute for Heart and Lung Research, 61231 Bad Nauheim, Germany; 6 International Laboratory EPIGEN, Consejo de Ciencia y Tecnología del Estado de Puebla (CONCYTEP), Universidad de la Salud del Estado de Puebla, Puebla 72000, Mexico; 7 Gastroenterology and Endoscopy, IRCCS Ospedale San Raffaele and University Vita-Salute San Raffaele, 20132 Milan, Italy; sdanese@hotmail.com

**Keywords:** inflammatory bowel disease, epigenetics, exposome

## Abstract

Inflammatory bowel diseases (IBD) are chronic inflammatory disorders of the gastrointestinal tract that encompass two main phenotypes, namely Crohn’s disease and ulcerative colitis. These conditions occur in genetically predisposed individuals in response to environmental factors. Epigenetics, acting by DNA methylation, post-translational histones modifications or by non-coding RNAs, could explain how the exposome (or all environmental influences over the life course, from conception to death) could influence the gene expression to contribute to intestinal inflammation. We performed a scoping search using Medline to identify all the elements of the exposome that may play a role in intestinal inflammation through epigenetic modifications, as well as the underlying mechanisms. The environmental factors epigenetically influencing the occurrence of intestinal inflammation are the maternal lifestyle (mainly diet, the occurrence of infection during pregnancy and smoking); breastfeeding; microbiota; diet (including a low-fiber diet, high-fat diet and deficiency in micronutrients); smoking habits, vitamin D and drugs (e.g., IBD treatments, antibiotics and probiotics). Influenced by both microbiota and diet, short-chain fatty acids are gut microbiota-derived metabolites resulting from the anaerobic fermentation of non-digestible dietary fibers, playing an epigenetically mediated role in the integrity of the epithelial barrier and in the defense against invading microorganisms. Although the impact of some environmental factors has been identified, the exposome-induced epimutations in IBD remain a largely underexplored field. How these environmental exposures induce epigenetic modifications (in terms of duration, frequency and the timing at which they occur) and how other environmental factors associated with IBD modulate epigenetics deserve to be further investigated.

## 1. Introduction

Inflammatory bowel diseases (IBD) are chronic relapsing-remitting inflammatory disorders of the gastrointestinal tract encompassing two main phenotypes: Crohn’s disease (CD) and ulcerative colitis (UC). The pathogenesis of IBD is not fully understood to date, but the most commonly accepted hypothesis is an inappropriate gut mucosal immune response towards the constituents of the gut microbiota, which cross an impaired epithelial barrier, in genetically predisposed individuals and under the influence of environmental factors [1]. Epidemiological studies (such as those carried out on monozygotic twins [2] and immigrants [3]), as well as the increase over time of the CD and UC incidence and prevalence (while the human gene pool is the same as before) [4], are all arguments that emphasize the importance of environmental factors in the occurrence of these inflammatory diseases. Epigenetics is a branch of life science that studies mechanisms regulating DNA-dependent processes (e.g., transcription, replication, recombination, repair, etc.) without primarily involving the nucleotide sequence of the DNA but, rather, the structure of how DNA is packed in the cell nucleus (chromatin structure), which can be inherited by daughter cells after cell division. Epigenetic mechanisms, including DNA methylation, post-translational histones modifications and non-coding ribonucleic acids (ncRNAs) [5]^,^ [6], regulating gene expression provide plausible explanations for the influence of the environment on gene expression profiles that favor intestinal inflammation [7]. Supporting this line of ideas, DNA methylation profiles observed in older monozygous twins with different environmental histories shows that epigenetic imprinting occurs mainly during crucial periods of development, whereas epigenomic changes can also occur day after day and accumulate over time in response to the exposome [8,9,10].

The term exposome has been proposed to encompass all environmental influences over the life course, from conception to death, that may influence disease emergence and clinical outcomes [11,12]. External environmental factors influencing the occurrence of IBD include the maternal lifestyle and in utero events [13], breastfeeding [14], diet [7], smoking habits [15,16], drugs [16,17,18], physical activity [16], stress [19], appendicectomy [16], vitamin D/UV exposure [16], infections [20] and hygiene [21]. While it is possible that these different factors directly induce epigenetic changes in the host, it is also possible that they influence the microbiome, an internal component of the exposome, and contribute to the occurrence of IBD through the exposome–microbiome–epigenome axis [22].

The impact of exposomes on the epigenetics in IBD has been poorly studied and is probably underestimated. This review aims to identify all the elements of the exposome that may play a role in intestinal inflammation through epigenetic modifications, as well as the underlying mechanisms that may contribute to IBD pathophysiology.

## 2. Epigenetics in IBD

Epigenetic mechanisms of gene expressions are involved in the intestinal epithelium homeostasis and in the development and differentiation of the immune cells, as well as in the modulation of responses generated by the immune system to defend against potential pathogens [23]. These epigenetic changes are reversible [24]. The genomic DNA in the eukaryotic cell nucleus is organized into chromatin. Chromatin consists of nucleic acids (genomic DNA and different types of RNAs); histone proteins (H2A, H2B, H3, H4 and H1) and non-histone chromatin-associated proteins [5,25,26]. Nucleosomes constitute the functional and structural units of chromatin. A nucleosome is built by around 146 bp of genomic DNA surrounding a histone octamer, which consists of two H2A–H2B dimers and one (H3–H4)2 tetramer [27,28]. In other words, chromatin is the physiological template for all DNA-dependent biological processes, including transcription. This fact increases the complexity of transcription regulation, since it implies that the chromatin structure has to be dynamic to grant or block access of transcription regulators to their respective binding elements on the DNA and to the transcription machinery to the genomic information in the nucleotide sequence. The epigenetic mechanisms of transcriptional regulation involve DNA methylation, histone modifications, nucleosome remodeling, interaction with the nuclear matrix and regulation via long non-coding RNAs (lncRNAs) and micro RNAs (miR) [26,29,30,31]. These mechanisms of transcription regulation establish cell-specific, heritable patterns of differential gene expression and silencing from the same genome and allow the cells to change these gene expression signatures in response to stimuli, such as changing conditions due to their environment [32,33].

DNA methylation in eukaryotes refers to the covalent transfer of a methyl group (-CH3) to the carbon atom at position 5 of cytosine forming 5-methylcytosine (5mC), most frequently at the dinucleotide sequence CG (mCG) [31,34,35,36,37]. DNA regions that are ≥200 bp long and show a CG:GC ratio ≥ 0.6 are defined as a CpG island [38]. The presence of DNA methylation prevents transcription factors from reaching gene promoters and generally leads to gene silencing [39,40]. DNA methylation in eukaryotes is catalyzed by DNA methyltransferases (DNMTs): DNMT1, which maintains DNA methylation patterns (during DNA replication and cell division), and DNMT3A/3B, which are responsible for de novo methylating DNMTs (during development or differentiation) [41,42,43]. These enzymes transfer methyl groups from S-adenosyl-L-methionine (SAM) to the cytosine residues in DNA [44]. On the contrary, DNA demethylation is mediated by the ten-eleven translocation (TET) enzymes, which add a hydroxyl group onto the methyl group of 5mC to form 5hmC (5-hydroxymethyl cytosine) [45]. Compared to healthy subjects, IBD patients show DNA methylation changes both at the cell (mainly immune) level and at the tissue level [46,47,48,49,50,51,52]. These changes also differ between UC and CD patients and involve several loci responsible for the regulation of immune responses [46,47,48,49,50,51,52].

Another epigenetic mechanism of transcriptional regulation involves post-translational modifications of histone proteins (further referred to as histone modifications). Histone proteins (H1, H2A, H2B H3 and H4) are relatively small and basic proteins that are abundant in the cell nucleus and are an essential part of the nucleosome, as described above. Due to structural characteristics of the nucleosome, histone proteins can undergo post-translational modifications at their N-terminal tails, which include acetylation, methylation, phosphorylation, ubiquitination and sumoylation, among others [53,54,55,56]. While DNA methylation is relatively stable in somatic cells, histone modifications are more diverse and dynamic, changing rapidly during the course of the cell cycle [6,30,53,54]. Acetylation at specific amino acids of histones (e.g., histone 3 lysine 9 acetylation; H3K9Ac) is generally associated with active chromatin and is mediated by histone acetyltransferases (HAT) and removed by histone deacetylases (HDAC). Histone methylation also occurs at specific amino acids of histone proteins and can be associated with both the repression (e.g., H3 lysine 27 trimethylation; H3K27me3) and activation (e.g., H3 lysine 4 trimethylation; H3K4me3) of gene expressions. There is a variety of enzymes mediating histone methylation (histone methyltransferases; HMT) and histone demethylation [57,58]. Similarly, the reactions leading to other histone modifications are catalyzed by a broad spectrum of enzymes in a regulated manner. Several environmental agents induce changes in histone modifications, thereby leading to changes in gene expression signatures.

In addition to these mechanisms, epigenetic regulation can also involve ncRNA, which are RNAs not translated into proteins, including miRs and lncRNAs. If miRs have a length of 18–25 nucleotides, lncRNAs are over 200 bases long [59]. These nucleic acid molecules can regulate gene expressions by interfering with messenger RNA (mRNA) translations by degrading them or through interactions with protein complexes involved in the regulation of gene expression [59,60]. The ncRNAs are differentially expressed between the control and IBD subjects, and there is also a difference in expression between CD and UC patients [61,62,63]. In IBD, miRs are involved in the regulation of the intestinal mucosal barrier, T-cell differentiation, the Th17 signaling pathway and autophagy [63]. In UC patients, miR-21, miR-16 and let-7 expressions are significantly increased in inflamed mucosa, while miR-192, miR-375 and miR-422b expressions are significantly reduced [61]. In CD patients, miR-23b, miR-106 and miR-191 are significantly increased in the inflamed mucosa, while miR-19b and miR-629 expressions are significantly decreased [61].

All these epigenetic mechanisms contribute to the development, progression and maintenance of IBD. They are usually triggered by a range of environmental factors. Some authors have mentioned three critical periods during which the environment can favor the onset of the disease: (1) during the prenatal period (in response to the maternal lifestyle), (2) in the early postnatal period (during gut microbiota colonization) and (3) just before the disease onset [64]. This review aims to study the impact of the exposome on the epigenome in IBD.

## 3. Methods

To identify exposome elements that could impact the epigenetics of IBD, we performed a scoping search using Medline. We used the following Medical Subject Heading (MeSH) terms (‘epigenetics’ OR ‘epigenomics’ OR ‘DNA methylation’ OR ‘histone(s)’ OR ‘short noncoding RNA’ OR ‘long noncoding RNAs’ OR ‘microRNA’ OR ‘miR’ OR “miRNA”) AND (“Inflammatory bowel disease” OR “IBD” OR “intestinal inflammation” OR “Crohn’s disease” OR “ulcerative colitis” OR “colitis”). Secondary references of the retrieved articles were reviewed to identify publications not captured by the electronic search. We excluded articles not written in English and those related to colitis-associated cancer.

## 4. Results

### 4.1. Parental Exposition

Accumulating evidence has pointed out that in utero environmental exposure can influence the epigenetic programming of the offspring and have an impact on its fate, conditioning its health status or, on the contrary, its lifelong risk of inflammatory conditions [65,66,67,68]. This is explained by the fact that the occurrence of an epimutation in a stem cell during embryonic development is transmitted to all their daughter cells and affects many more cells than those occurring in adult stem and/or somatic cells during postnatal development [69]. These epigenetic changes can not only be transmitted during successive division but also are passed on from generation to generation, some authors mentioning a real transgenerational epigenetic inheritance [70,71,72,73,74,75,76,77,78,79,80]. These prenatal environmental induced-epigenetic modifications could therefore contribute to the IBD epidemic not only by contributing to this condition but also by passing on modifications to subsequent generations, contributing to familial IBD predisposition, as illustrated by immigration studies [64,81,82,83,84,85].

There are few data on prenatal epigenetic plasticity in response to the environment in intestinal inflammation [64]. Some data suggest that this epigenomic reprogramming occurs in response to maternal diet modifications, and an excess of prenatal micronutrients (i.e., methyl donors routinely incorporated into prenatal supplements, such as folate, methionine, betaine and vitamin B12) in the maternal diet could confer an increased risk of colitis in the offspring [73]. The occurrence of maternal infection during pregnancy could also lead to the production of IL-6, known to induce epigenetic changes in fetal intestinal epithelial stem cells, which could induce long-lasting impacts on intestinal immune homeostasis and a predisposition toward inflammatory disorders [86]. In addition to diet and infections, maternal smoking during pregnancy could also have an impact on the risk of developing IBD [87]. A study of the impact of prenatal maternal smoking on the offspring’s DNA methylation has made it possible to highlight 69 differentially methylated CpGs in 36 genomic regions, among which four CpG sites were associated with an increased risk of IBD [87]. Maternal smoking induced persistent alterations in DNA methylation (rather, global hypomethylation [88,89,90,91,92]) but also miR dysregulation in the exposed offspring, changes that can be transmitted to the next generation [90,93,94,95,96,97,98]. Taken together, these data suggest that these maternal influences during prenatal development can induce epigenetic changes in the offspring, sometimes considered by some authors as the first step towards IBD development (by introducing a permanent change in the disease-relevant cell types) [64,99,100].

### 4.2. Microbiota

Occurring in this predisposing environment, a microorganism’s gut colonization during the first hours of life can be considered as the second step toward the occurrence of IBD [64,99,100]. Influenced by the mode of delivery, the presence or absence of breastfeeding and early environmental exposure, the early-life gut microbiota sets trajectories for health or IBD [101,102]. This newly formed microbiome will modulate until the age of 3 years to reach a globally largely similar taxonomic composition as in adults and will act as an epigenetic modulator, modifying the epimutations induced in the prenatal period. Breastfeeding and early bacterial colonization appear to play an important role in DNA methylation in intestinal epithelial stem cells and to condition the lifelong gut health [103].

The microbiome can induce epigenetic changes both in the intestinal epithelium and in immune cells (Table 1). Comparing the epigenomes of germ-free mice or antibiotic-treated mice to conventional mice, it appears that this microbiome can influence the host epigenetics through changes in DNA methylation, histone modifications and, also, through ncRNAs [104,105,106,107,108]. Species belonging to Firmicutes (especially *Faecalibacterium prausnitzii* and *Roseburia* species [109]) and Bacteroides genera, known to be reduced in IBD [110], have an epigenetically mediated anti-inflammatory action (HDAC inhibition) via the production of short-chain fatty acids (SCFAs) (the role of these will be discussed in more detail below) [111]. The commensal flora can also affect the bioavailability of methyl groups through their production of folate and affects the host DNA methylation [112,113].

Some germs may also contribute to the occurrence of IBD through their epigenetic mechanisms. Adherent-invasive *Escherichia coli* (AIEC), commonly associated with CD [114], upregulates the levels of miR-30c and miR-130a in intestinal epithelial cells (IECs), which reduces the levels of ATG5 and ATG16L1 and inhibits autophagy, leading to increased numbers of intracellular AIEC and the inflammatory response [115]. In turn, AIEC-infected IECs secrete exosomes that can transfer these same miR to recipient IECs with the same consequences, promoting the invasion and proliferation of infected tissues [116]. In addition, AIEC triggers an excessive mucosal immune response against the gut microbiota via the let-7b/TLR4 miR signaling pathway [117]. *Mycobacterium avium* subspecies *paratuberculosis* (MAP), also known to be associated with IBD [118], induces miR-21 expression in infected macrophages and decreases their ability to eliminate the bacteria, thus contributing to intestinal inflammation [119].

Microbial components such as lipopolysaccharides (LPS) and flagellin may also induce host epigenetic changes. LPS (a major component of the Gram-negative bacteria outer membrane) contributes to the development of intestinal inflammation by promoting the activation of NF-κB (nuclear factor-kappa B) pathways and the cytokines released by the downregulation of miR-19b, miR-497 and miR-215 in IECs [120], monocyte/macrophage cells [121] and fibroblast cells [122], respectively. LPS can also increase the level of H19 lncRNA in IECs that bind to miR (miR-34a and let-7), inhibiting cell proliferation and, thus, impairing the intestinal epithelial barrier [20]. In contrast, the flagellins of some bacteria—in particular, *Roseburia intestinalis* (found in a reduced abundance in IBD patients)—have rather epigenetically mediated anti-inflammatory actions [123,124]. Flagellin inhibits the activation of the NLRP3 (NOD-like receptor family, pyrin domain containing 3) inflammasome and proptosis in macrophages via miR-223-3p [123] and induces a lncRNA (HIF1A-AS2) that inactivates the NF-κB/Jnk (c-Jun N-terminal kinase) pathway [124].

**Table 1:** **Impact of the microbiota on the epigenome in intestinal inflammation**. AIEC, adherent-invasive Escherichia coli; CpG, cytosine–phosphate–guanine; DCs, dendritic cells; DNA, deoxyribonucleic acid; DNMT, DNA methyltransferase; DSS, dextran sulfate sodium; ETBF, Enterotoxigenic Bacteroides fragilis; HDAC, histone deacetylases; IECs, intestinal epithelial cells; IL, interleukin; KO, knockout; LPS, lipopolysaccharide; lncRNAs, long non-coding RNAs; MAP, Mycobacterium avium subspecies paratuberculosis; miR, micro-RNA; NF-κB, nuclear factor-kappa B; NLRP3, NOD-like receptor family pyrin domain containing 3; NOD2, nucleotide-binding oligomerization domain 2; PSC, primary sclerosing cholangitis; STAT, signal transducer and activator of transcription; TLR, toll-like receptor; TNBS, 2,4,6-trinitrobenzenesulfonic acid; UC, ulcerative colitis; WT, wild-type; ↑, increase; ↓ decrease.

Germ	Activity	Epigenetic Mechanism	Tissue/Cells	Mechanism	Model	Author
**Commensal bacteria**						
Commensal bacteria	Anti-inflammatory activity	miR-10a	DCs	Negatively regulates host miR-10a expression, which contribute to the intestinal homeostasis maintenance by targeting IL-12/IL-23p40 expression	C57BL/6 (B6) mice	Xue X, et al. (2011) [125]
Commensal flora	Proinflammatory activity	miR-107	DCs and macrophages	Downregulates miR-107 expression, known to represses the expression of IL-23p19, thereby favouring IL-23 expression	IECs, lamina propria CD11c+ myeloid cells including dendritic cells and macrophages, and T cells; DSS-induced colitis in mice	Xue X, et al. (2014) [126]
Commensal bacteria	Anti-inflammatory activity	miR-10a	DCs	Inhibits human DCs miR-10a expression, which downregulates mucosal inflammatory response through inhibition of IL-12/IL-23p40 and NOD2 expression, and blockade of Th1/Th17 cell immune responses	Human monocyte-derived dendritic cells	Wu W, et al. (2015) [127]
Commensal microbiome-dependent (*Bacteroides acidifaciens* and *Lactobacillus johnsonii*	Anti-inflammatory activity	miR-21-5p	IECs	Commensal microbiome-dependent miR-21-5p expression in IECs regulates intestinal epithelial permeability via ADP Ribosylation Factor 4 (ARF4)	HT-29 and Caco-2 cells	Nakata K., et al. (2017) [128]
**Cluster(s)**						
↓ of Bacteroidetes and ↑ of protective Firmicutes and *Clostridia*	Anti-inflammatory activity	miR-21	Colonic mucosae	Leads to miR-21 reduction, known to influence the pathogenesis of intestinal inflammation by causing propagation of a disrupted gut microbiota	WT and miR-21^−/−^ mice	Johnston DGW, et al. (2018) [129]
Cluster enriched in *Bacteroides fragilis*	-	DNA methylation	Intestinal mucosa	Induces 33 and 19 significantly hyper-methylated or hypomethylated sites, including hyper-methylated signals in the gene body of Notch Receptor 4 (NOTCH4)	50 CD; 80 UC; 31 controls	Ryan FJ, et al. (2020) [130]
Cluster enriched in *Escherichia*/*Shigella*/*Klebsiella* and *Ruminococcus gnavus*	Proinflammatory activity	DNA methylation	Intestinal mucosa	Larger number of differentially methylated CpG sites (131 hyper- and 475 hypomethylated), including hypomethylation in CCDC88B (recently correlated with risk of CD) and Transporter 2 (TAP2), involved in genetic heterogeneity of CD		
Cluster enriched in *B. vulgatus*	-	DNA methylation	Intestinal mucosa	Induces 23 hyper- and 18 hypomethylated sites, significant hyper-methylation was observed in the gene body of DNA Damage Regulated Autophagy Modulator 1 (DRAM1)		
**Specific germ**						
*Adherent-invasive Escherichia coli (AIEC)*	Proinflammatory activity	miR-30c and miR-130a	IECs	Upregulates levels of miR-30c and miR-130a in IECs (by activating NF-κB), reducing the levels of ATG5 and ATG16L1 and inhibiting autophagy, leading to increased numbers of intracellular AIEC and an increased inflammatory response	Cultured IECs and mouse enterocytes	Nguyen HT, et al. (2014) [115]
*AIEC*	Proinflammatory activity	let-7b	IECs	Instigates excessive mucosal immune response against gut microbiota via miR let-7b/TLR4 signaling pathway	WT and IL-10 KO mice; T84 cells	Guo Z, et al. (2018) [117]
*AIEC*	Proinflammatory activity	miR-30c and miR-130a	IECs	*AIEC*-infected IECs secretes exosomes that can transfer specific miRs (miR-30c and miR-130a) to recipient IECs, inhibiting autophagy-mediated clearance of intracellular *AIEC*	T84 cells	Larabi A, et al. (2020) [116]
*Mycobacterium avium subspecies paratuberculosis (MAP)*	Proinflammatory activity	miR-21	Macrophages	MAP upregulates miR-21 in macrophages, a change that results in diminished macrophages clearance ability and favours pathogens survival within the cells	THP-1 cells	Mostoufi-Afshar S, et al. (2018) [119]
*Lactobacillus rhamnosus GG*	Anti-inflammatory activity	miR-146a and miR-155	DCs	Induces a significant downregulation of miR-146a expression, a negative regulator of immune response, and upupregulation of on miR-155	Cultured DCs	Giahi L., et al. (2012)
*Lactobacillus acidophilus*	Anti-inflammatory activity	miRs	Colonic mucosae	*L. acidophilus* induce miRs expression	DSS-induced colitis in mice	Kim WK, et al. (2021) [131]
Faecalibacterium prausnitzii	Anti-inflammatory activity	HDAC1 inhibition	T cells	Inhibits HDAC1, promotes Foxp3 and blocks the IL-6/STAT3/IL-17 downstream pathway contributing to the maintain of Th17/Treg balance	IBD patients (*n* = 9) and healthy control (*n* = 6); DSS-induced colitis in mice	Zhou L, et al. (2018) [132]
Faecalibacterium prausnitzii	Anti-inflammatory activity	HDAC3 inhibition	T cells	Produces butyrate to decrease Th17 differentiation and attenuate colitis through inhibiting HDAC3 and c-Myc-related metabolism in T cells	IBD patients; TNBS-induced colitis in mice	Zhang M, et al. (2019) [133]
*Trichinella spiralis*	Anti-inflammatory activity	miRs	T cells	Extra-vesicles-derived miR are involved in the regulation of the host immune response, including inflammation, including increase of Th2 and Treg cells	TNBS-induced colitis in mice	Yang Y, et al. (2020) [134]
*Enterotoxigenic Bacteroides fragilis (ETBF)*	Proinflammatory activity	miR-149-3p	T cells	Downregulates miR-149-3p, which play a role in modulation of T-helper type 17 cell differentiation (with increased number of T-helper type 17 cell contributing to intestinal inflammation)	ETBF cells	Cao Y, et al. (2021) [135]
**Bacterial component**						
*Roseburia* intestinalis-derived flagellin	Anti-inflammatory activity	lncRNA	IECs	Flagellin induces p38-stat1 activation, activated HIF1A-AS2 promotor, induced HIF1A-AS2 (a lncRNA) expression in gut epithelium in a dose- and time-dependent manner. HIF1A-AS2 inactivates NF-κB/Jnk pathway and thus inhibits inflammatory responses	DSS/Flagellin-challenged mice; Caco-2 cells	Quan Y, et al. (2018) [124]
*Roseburia* intestinalis-derived flagellin	Anti-inflammatory activity	miR-223-3p	Macrophages	Flagellin inhibited activation of the NLRP3 inflammasome and pyroptosis via miR-223-3p/NLRP3 signaling in macrophages	DSS-induced colitis model in C57Bl/6 mice and the LPS/ATP-induced THP-1 macrophages	Wu X, et al. (2020) [123]
LPS	Proinflammatory activity	H3K4me1, H3K4me3, and H3K27ac histone	Macrophages	Increases H3K4me1, H3K4me3, and H3K27ac histone marks, particularly in genes associated with an inflammatory response such as IL-12a and IL-18	IL-10-deficient (Il10^(−/−)^) mice	Simon JM, et al. (2016) [136]
LPS and flagellin	Anti-inflammatory activity	miR-146	IECs	Stimulate miR-146a overexpression in IECs, induces immune tolerance, inhibiting cytokine production (MCP-1 and GROα/IL-8)	TNBS and DSS-induced colitis in mice	Anzola A, et al. (2018) [137]
LPS	Proinflammatory activity	lncRNA H19	IECs	Increases levels of H19 lncRNA in epithelial cells in the intestine. H19 lncRNA bound to p53 and miR (miR-34a and let-7) that inhibit cell proliferation (alters regeneration of the epithelium)	Intestinal tissues of UC patients and mice	Geng H, et al. (2018) [138]
LPS	Proinflammatory activity	miR-19b	IECs	LPS significantly induces cell inflammatory injury, downregulated miR-19b expression and activates NF-κB and PI3K/AKT pathway	Caco2 cells	Qiao CX, et al. (2018) [120]
LPS	Proinflammatory activity	lncRNA	Monocytes/macrophages	LPS promotes a downregulation of the lncRNA growth arrest-specific transcript 5 (*GAS5*), could mediate tissue damage by modulating the expression of matrix metalloproteinases	IBD patients (*n* = 25)	Lucafò M, et al. (2019) [139]
LPS	Proinflammatory activity	miR-215	Fibroblasts	LPS upregulates the expression of miR-215, increases oxidative stress in LPS-treated intestinal fibroblast by downregulating GDF11 (Growth differentiation factor 11) expression and activating the TLR4/NF-κB and JNK/p38 signaling pathways	CCD-18Co cells	Sun B, et al. (2020) [122]
LPS	Proinflammatory activity	miR-506 and DNMT1 modification	IECs	LPS inhibits miR-506, leading to reduced expression of anion exchange protein 2 and inositol-1,4,5-trisphosphate-receptor but was accompanied by a substantial increase in DNMT1 and SPHK1 (sphingosine kinase 1) expression. The enhanced levels of kinase SPHK1 resulte in upregulation of bioactive sphingosine-1-phosphate (S1P) which led to further activation of S1P-dependent signaling pathways. The net effect of these responses is severe inflammation	Patients with PSC, PSC with concurrent UC (PSC + UC), UC alone, and healthy controls (*n* = 10 each); Caco2 cells	Kempinska-Podhorodecka A, et al. (2021) [140]
LPS	Proinflammatory activity	miR-497	Macrophages	Reduces miR-497, promotes the activation of NF-κB pathway and the release of cytokines	IBD patients, mice with colitis and LPS-treated RAW264.7 cells	Zhang M, et al. (2021) [121]

Although the taxonomic composition of the microbiota is stable at year 3, its composition can be influenced by a range of other environmental factors (including dietary habits, smoking and drugs, as discussed below), which may be responsible for the third step towards the occurrence of IBD [64].

### 4.3. Gut Microbiota-Derived Metabolites

Influenced by both the microbiota and diet, SCFAs are gut microbiota-derived metabolites that result from the anaerobic fermentation of nondigestible dietary fibers (found in fruits and vegetables). Acetate, butyrate and propionate, the three principal SCFAs, exert an anti-inflammatory role and promote the integrity of the epithelial barrier functions partly via the epigenetic pathways (Table 2) [141,142]. Among the SCFAs, butyrate is the most studied one. By inhibiting, in a reversible way, HDACs [143,144], cells exposed to butyrate present higher acetylation at specific lysine residues in histones, resulting in increased transcription of genes in both intestinal epithelial and immune cells [145]. The inhibition of HDAC in cells contributes to the reduction of inflammation by (1) the induction of IκBα expression, with a subsequent inhibition of the NF-κB pathway, (2) the inhibition of the IFN-γ/STAT1 (signal transducer and activator of transcription) signaling pathway and (3) the activation of the anti-inflammatory function of PPARγ (peroxisome proliferator-activated receptor γ) [145]. Butyrate also has more specific epigenetic actions on certain cell types. At the epithelial level, butyrate plays a role in the integrity of the epithelial barrier (by restoring tight junction proteins [146]) and the defense against the invading microorganisms (via a nucleotide-binding oligomerization domain 2 (NOD2)-dependent pathway or via autophagy [147]). Butyrate also has an effect on various immune cells, such as (1) monocytes/macrophages (in which it induces monocyte-to-macrophage differentiation, promotes their antimicrobial activity through inhibition of HDAC3 [148], reduces the production of their inflammatory mediators [149] and induces the polarization of M2 macrophages [150]); (2) T cells (promotes Treg [151] and inhibits Th17 cell development [151]); (3) neutrophils (in which HDAC inhibition leads to proinflammatory cytokine reduction [152]) and (4) dendritic cells (inhibit IL-12 [153]). The epigenetic role of propionate and acetate has been less studied. Propionate promotes epithelial cell migration and contributes to intestinal epithelial restitution, a complex process important for tissue regeneration in IBD [142].

### 4.4. Diet

Next, compared to a low-fiber diet [163], impacting the level of these SCFAs [163], other diets have been shown to induce epigenetic changes related to IBD (Table 3). Regarding the literature, elements of the Western diet, characterized by a low-fiber, low-fruit, low-vegetable and deficiency in micronutrients, as well a high-fat diet, may be associated with epigenetic changes in IBD. The Western diet has been shown to lead to a decrease in miR-143/145a, miR-148a and miR-152 in colonocytes with a consequent increase in ADAM17 (a disintegrin and metalloprotease 17) expression protein and colitis aggravation [164]. A low or deficient methyl diet can also contribute to intestinal inflammation by reducing SIRT1 (sirtuin 1) expression (a histone deacetylase), contributing to endoplasmic reticulum stress [165] and demethylating HIF-1-responsive elements (HRE), which leads to the abnormal gut expression of CEACAM6 (CEA Cell Adhesion Molecule 6), favoring AIEC colonization and subsequent inflammation [166]. Finally, it was shown that a high-fat diet can change the miR profile of the visceral adipose exosomes (switching the exosomes from an anti-inflammatory to a proinflammatory phenotype with an increase of miR-155, for example), predisposing the intestine to inflammation via promoting macrophage M1 polarization [167].

Polyphenols, found mainly in fruits and vegetables, are complex molecules produced by plants with antioxidant properties able to scavenge free radicals. Divided into flavonoids (such as alpinetin, fortunellin, baicalin, quercetin, berberine, cardamonin and lonicerin) [168,169,170,171,172,173,174] and non-flavonoids (such as resveratrol [175,176] and chlorogenic acid [177]), they reduce the risk of intestinal inflammation, mainly by modifying the miRs profile and inhibiting HDACs. Other foods have also been shown to influence host epigenetics and could potentially play a role in gut inflammation. Milk [178,179,180], common sweeteners [181], galacto-oligosaccharides [182], corn cobs [183], cinnamaldehyde (a major active compound from cinnamon) [184,185], limonin (a triterpenoid extracted from citrus) [186], ginger [187], ginseng [188] and black raspberries [189,190] have anti-inflammatory properties. In contrast, chronic alcohol exposure increases miR-122a and miR-155 expression in the intestine, which decreases occludins expression, leading to increased intestinal permeability and modulates cytokines and the T-cell immune response in the gut, leading to intestinal TNFα (tumor necrosis factor α) and NF-κB activation, respectively [191,192].

**Table 3:** **Impact of the diet on the epigenome in intestinal inflammation**. ADAM17, a disintegrin and metalloprotease-17; AIEC, Adherent-invasive Escherichia coli; CD, Crohn’s disease; CEACAM6, carcinoembryonic antigen-related cell adhesion molecule 6; CREB, C-AMP response element-binding protein; DNA, deoxyribonucleic acid; DNMT, DNA methyltransferase; DSS, dextran sulfate sodium; HDAC, histone deacetylase; HMGB1, high mobility group box 1; HRE, HIF-1-responsive elements; IBD, inflammatory bowel disease; ICAM, intercellular adhesion molecule; IEC, intestinal epithelial cell; IFN, interferon; IL, interleukin; lncRNA, long non-coding RNA; LPS, lipopolysaccharide; miR, microRNA; MMP9, Matrix Metallopeptidase 9; PBMC, peripheral blood mononuclear cell; PTEN, phosphatase and tensin homolog; NF-κB, nuclear factor-kappa B; NLRP3, NOD-like receptor family pyrin domain containing 3; PECAM, Platelet endothelial cell adhesion molecule; RECK, reversion-inducing cysteine-rich protein with Kazal motifs; SCFAs, short-chain fatty acids; STAT, signal transducer and activator of transcription; TGF, transforming growth factor; TNBS, 2,4,6-trinitrobenzenesulfonic acid; TNF, tumor necrosis factor; UC, ulcerative colitis; VCAM, vascular cell adhesion molecule 1; WT, wild-type; ZO, zonula occludens.

Food	Activity	Epigenetic Mechanism	Tissue/Cells	Mechanism	Model	Author
**Diet**						
Western diet	Proinflammatory activity	miR-143, miR-145A, miR-148a, miR-152	IECs	Leads to a decrease in miR-143/145a, miR-148a and miR-152 in colonocytes with a consequent increase in ADAM17 expression protein (these miRs regulating ADAM17) and aggravates colitis.	DSS-induced colitis in mice	Dougherty U, et al. (2021) [164]
High fat diet	Proinflammatory activity	miR-155	Visceral adipocytes	High fat diet changes the miR profile (among which miR-155) of the visceral adipose exosomes, switching the exosomes from anti-inflammatory to a proinflammatory phenotype.	Macrophages	Wei M, et al. (2020) [193]
High fat diet rich in n-6 linoleic acid	Proinflammatory activity	DNA methylation	Colonic mucosae	Epigenetically modifies farnesoid-X-receptor (*FXR*), leading to the activation of downstream factors that participate in bile acid homeostasis and epigenetically activates prostaglandin-endoperoxide synthase-2 (Ptsg-2) coupled accumulation of c-JUN and proliferative cyclin D1(Ccnd1) and increase the risk of inflammation	C57BL/6J mice; Human colonic foetal cells	Romagnolo DF, et al. (2019) [194]
Methyl-deficient diet	Proinflammatory activity	Sirtuin 1	IECs	Reduces sirtuin 1 (SIRT1) expression level and promotes greater acetylation of (heat shock factor protein 1) HSF1, in relation with a dramatic decrease of chaperones (binding immunoglobulin protein (BIP), heat shock protein (HSP)27 and HSP90)	DSS-induced colitis in mice; Caco-2 cells	Melhem H, et al. (2016) [165]
Low-methyl diet	Proinflammatory activity	DNA methylation	IECs	Low-methyl diet-dependent HRE demethylation led to abnormal gut expression of CEACAM6 (carcinoembryonic antigen-related cell adhesion molecule 6), favouring AIEC colonisation and subsequent inflammation	Transgenic mice; Caco-2, T-84 and sh-HIF1-α-T-84 cells	Denizot J, et al. (2015) [166]
Methyl-donnor supplemented diet (folate, B12 vitamin)	Anti-inflammatory activity	DNA methylation	IECs	Methyl-donor supplemented diet contributes to hypermethylation of CEACAM6 promoter in IECs, associated with a significant decrease in CEACAM6 expression contributing to less adherence of AIEC bacteria to the enterocytes	CEABAC10 mice	Gimier E, et al. (2020) [195]
**Isolated food**						
Cow’s milk (commercial)	Anti-inflammatory activity	miR-21, miR-29b and miR-125b	Colonic mucosae	Extracellular vesicles (EVs) concentrated from commercial cow’s milk downregulates miR-21, miR-29b and miR-125b. MiR-125b was associated with a higher expression of the NF-κB inhibitor TNFAIP3 (A20)	DSS-induced colitis in mice	Benmoussa A, et al. (2019) [178]
Human milk derived exosomes	Anti-inflammatory activity	miR-320, miR-375, and Let-7 and DNMT1 and DNMT3	Colonic mucosae	MiR highly express in milk, such as miR-320, 375, and Let-7, were found to be more abundant in the colon of milk derived exosomes-treated mice compared with untreated mice. These miR downregulate their target genes, mainly DNA methyltransferase 1 (DNMT1) and DNMT3	DSS-induced colitis in mice; PBMC	Reif S, et al. (2020) [179]
Dietary depletion of milk exosomes and their microRNA cargos	Proinflammatory activity	miR-200a-3p	Cecum mucosae	Elicits a depletion of miR-200a-3p and elevated intestinal inflammation and chemokine (C-X-C Motif) ligand 9 expression	Mdr1a^−/−^ mice	Wu D, et al. (2019) [180]
Saccharin sodium, Stevioside, and Sucralose (three common sweeteners)	Anti-inflammatory activity	miR-15b	IECs	Upregulate the expression of E-cadherin through the miR-15b/RECK/MMP-9 axis to improve intestinal barrier integrity. Saccharin exerts the most pronounced effect, followed by Stevioside and Sucralose	DSS-induced colitis in mice	Zhang X, et al. (2022) [181]
Galacto-oligosaccharides (GOS)	Anti-inflammatory activity	miR-19	IECs	GOS increases of cell viability, the decrease of apoptosis, as well as the suppressed release of TNF-α, IFN-γ and IL-1β by upregulating miR-19b	Human colon epithelial FHC cells; Helicobacter hepaticus induced colitis in rats	Sun J, et al. (2019) [182]
Cinnamaldehyde (a major active compound from cinnamon)	Anti-inflammatory activity	miR-21 and miR-155	Macrophages	Cinnamaldehyde inhibits NLRP3 inflammasome activation as well as miR-21 and miR-155 level in colon tissues and macrophage. The decrease in miR-21 and miR-155 suppresses levels of IL-1β and IL-6;	DSS-induced colitis in mice; macrophage cell line RAW264.7 and human monocytes U937	Qu S, et al. (2018) [185]
Cinnamaldehyde	Anti-inflammatory activity	lncRNAs H19	T cells	Cinnamaldehyde inhibits Th17 cell differentiation by regulating the expression of lncRNA H19	DSS-induced colitis in mice and naïve CD4+ T cells	Qu SL, et al. (2021) [101]
Limonin (a triterpenoid extracted from citrus)	Anti-inflammatory activity	miR-124	IECs	Downregulates p-STAT3/miR-214 signaling pathway and represses the productions of proinflammatory cytokines (such as TNF-α and IL-6)	DSS-induced colitis in mice; cultured normal colonic epithelial cells	Liu S, et al. (2019) [186]
Edible ginger	Anti-inflammatory activity	Contained around 125 miRNAs	IECs	Increases the survival and proliferation of IECs, reduces the proinflammatory cytokines (such as TNF-α, IL-6 and IL-1β), and increases the anti-inflammatory cytokines (including IL-10 and IL-22) in colitis	DSS-induced colitis in mice	Zhang M, et al. (2016) [187]
Ginsenoside Rh2 (active ingredient of ginseng)	Anti-inflammatory activity	miR-124	IECs	Inhibits IL-6-induced STAT3 phosphorylation and miR-214 expression (which is an inflammatory effector molecule acting through NF-κB-IL6 pathway)	DSS-induced colitis in mice; cultured normal colonic epithelial cells	Chen X, et al. (2021) [188]
Black raspberries (BRBs)	Anti-inflammatory activity	Demethylation the promoter of dkk3; correction of promoter hypermethylation of suppressor genes	Colonic mucosae	BRBs exert their anti-inflammatory effects is through decreasing NF-κB p65 expression leading to decrease of DNMT3B expression (but also histone deacetylases 1 and 2 (HDAC1 and HDAC2) and methyl-binding domain 2 or MBD2), which in turn reverse aberrant DNA methylation of tumor suppressor genes, e.g., dkk2, dkk3, in the Wnt pathway, resulting in their enhanced mRNA expression locally in colon and systematically in spleen and bone marrow and thus in decreased translocation of β-catenin to the nucleus prohibiting the activation of the pathway	DSS-induced colitis in mice; splenocytes and bone marrow cells	Wang LS, et al. (2013) [189]
Black raspberries	Anti-inflammatory activity	Demethylation	Colonic mucosae	BRBs decreas the methylation of wif1, sox17, and qki gene promoters and thus increase their mRNA expression (contributing to Wnt signaling)	Interleukin-10 knockout mice	Wang LS, et al. (2013) [190]
Mastiha	Anti-inflammatory activity	miR-155	T cells	Plays a role in circulating levels of miR-155, a critical player in T helper-17 (Th17) differentiation and function	UC patients (*n* = 35)	Amerikanou C, et al. (2021) [196]
Isoliquiritigenin	Anti-inflammatory activity	HDACs inhibition	IECs	Suppresses acetylated HMGB1 release via the induction of HDAC activity, which is one of the critical mediators of inflammation, which is actively secreted from inflammatory cytokine-stimulated immune or non-immune cells	HT-29 cells	Chi JH, et al. (2017) [197]
Chronic ethanol exposure	Proinflammatory activity	miR-122a	IECs	Increases the intestinal miR-122a expression, which decreased occludin (OCLN) expression leading to increased intestinal permeability	HT-29 cells	Chen Y, et al. (2013) [191]
Chronic alcohol feeding (but not acute alcohol binge)	Proinflammatory activity	miR-155	Intestinal tissue	Increases miR-155 in the small bowel, which is a modulator of cytokine and T-cell immune response in the gut, leading to intestinal TNFα, and NF-κB activation	WT-mice	Lippai D, et al. (2014) [192]
**Polyphenol**						
Polyphenolic red wine extract	Anti-inflammatory activity	miR-126	Fibroblasts	Polyphenolic red wine extract downregulates miR-126, leading to downregulation of NF-kB, ICAM-1, VCAM-1, and PECAM-1	CCD-18Co myofibroblasts cells	Angel-Morales G, et al. (2012) [198]
Polyphenolic extracts from cowpea (*Vigna unguiculata*)	Anti-inflammatory activity	miR-126	Fibroblasts	Cowpea may exert their anti-inflammatory activities at least in part through induction of miR-126 that then downregulate VCAM-1 mRNA and protein expressions	CCD-18Co myofibroblasts cells	Ojwang LO, et al. (2015) [199]
Mango (*Mangifera indica* L.) polyphenolics	Anti-inflammatory activity	miR-126	Fibroblasts	Mango polyphenols attenuates inflammatory response by modulating the PI3K/AKT/mTOR pathway at least in part through upregulation of miR-126 expression	CCD-18Co cells; DSS-induced colitis in rats	Kim H, et al. (2017) [200]
Baicalin (flavone)	Anti-inflammatory activity	miR-191a	IECs	Exerts a protective effect on IECs against TNF-α-induced injury, which is at least partly via inhibiting the expression of miR-191a, thus increasing ZO-1 expression	IEC-6 cells	Wang L, et al. (2017) [170]
Pomegranate (*Punica granatum* L.) polyphenolics	Anti-inflammatory activity	miR-145	Myofibroblasts	Pomegranate polyphenols attenuate colitis by modulating the miR-145/p70S6K/HIF1α axis	DSS-induced colitis in rats; CCD-18Co colon-myofibroblastic cells	Kim H, et al. (2017) [201]
Alpinetin, a flavonoid compound extracted from the seeds of *Alpinia katsumadai* Hayata	Anti-inflammatory activity	miR-302	T cells	Activates Aryl hydrocarbon receptor (AhR), promoting expression of miR-302, downregulating expression of DNA methyltransferase 1 (DNMT-1), reducing methylation level of Foxp3 promoter region, facilitating combination of CREB and promoter region of Foxp3, and upregulating the expression of Foxp3. Alpinetin ameliorates colitis in mice by recovering Th17/Treg balance.	DSS-induced colitis in mice	Lv Q, et al. (2018) [168]
Fortunellin, a citrus flavonoid	Anti-inflammatory activity	miR-374a	IECs	Fortunellin targets miR-374a, which is a negative regulator of PTEN, known to induce cell apoptosis	TNBS-induced colitis in rats	Xiong Y, et al. (2018) [169]
Quercetin (flavonoid)	Anti-inflammatory activity	miR-369-3p	DCs	Quercetin-induced miR-369-3p which reduce C/EBP-β, TNF-α, and IL-6 production	LPS-stimulated DCs	Galleggiante V, et al. (2019) [171]
Resveratrol (a natural plant product)	Anti-inflammatory activity	miR-31, Let7a, miR-132	T cells	Resveratrol decreases the expression of several miRs (miR-31, Let7a, miR-132) that targets cytokines and transcription factors involved in anti-inflammatory T cell responses (Foxp3 and TGF-β). MiR-31 regulates the expression of Foxp3 with increase of CD4+ Foxp3+ regulatory T cells (Tregs)	TNBS-induced colitis in mice	Alrafas HR, et al. (2020) [176]
Resveratrol (an anti-oxidant)	Anti-inflammatory activity	HDACs inhibition	T cells	Inhibits HDACs, increases anti-inflammatory CD4+ FOXP3+ (Tregs) and CD4+ IL10+ cells, and decreases proinflammatory Th1 and Th17 cells	AOM and DSS-induced colitis in mice	Alrafas HR, et al. (2020) [175]
Chlorogenic acid (found in the coffee)	Anti-inflammatory activity	miR-155	Macrophages	Downregulates miR-155 expression, inactivates the NF-κB/NLRP3 inflammasome pathway in macrophages and prevent colitis	DSS-induced colitis in mice; LPS/ATP-induced RAW264.7 cells	Zeng J, et al. (2020) [177]
Lonicerin (constituant of herb *Lonicera japonica* Thunb.)	Anti-inflammatory activity	H3K27me3 modification	Macrophages	Binds to enhancer of zeste homolog 2 (EZH2) histone methyltransferase, which mediate modification of H3K27me3 and promotes the expression of autophagy-related protein 5, which in turn leads to enhanced autophagy and accelerates autolysosome-mediated NLRP3 degradation	DSS-induced colitis in mice and isolated colonic macrophages and IECs; bone marrow-derived macrophages	Lv Q, et al. (2021) [174]
Pristimerin (Pris), which is a natural triterpenoid compound extracted from the *Celastraceae plant*	Anti-inflammatory activity	miR-155	Colonic mucosae	Pris may reduce DSS-induced colitis in mice by inhibiting the expression of miR-155	Blood and colon tissue of IBD patients; DSS-induced colitis in mice	Tian M, et al. (2021) [202]
Cardamonin is a naturally occurring chalcone (majorly from the *Zingiberaceae family* incluging a wide range of spices from India)	Anti-inflammatory activity	Modulation of miR expression	Macrophages	Cardamonin modulates miR expression, protects the mice from DSS-induced colitis, decreases the expression of iNOS, TNF-α, and IL-6, and inhibited NF-kB signaling which emphasizes the role of cardamonin as an anti-inflammatory molecule	RAW 264.7 Cells (monocyte/macrophage-like cells); DSS-induced colitis in mice	James S, et al. (2021) [172]
Berberine	Anti-inflammatory activity	miR-103a-3p	IECs	Represses Wnt/β-catenin pathway activation via modulating the miR-103a-3p/Bromodomain-containing protein 4 axis, thereby refraining pyroptosis and reducing the intestinal mucosal barrier defect induced via colitis	DSS-induced colitis in mice; Caco-2 cells and human NCM460 cells	Zhao X, et al. (2022) [173]

### 4.5. Smoking

Smoking habits are the single best-established environmental factor that influences the CD phenotype, behavior and response to therapy [203]. While nicotine is the most prominent component released during smoking (and therefore the best-studied), other chemical components could also induce epigenetic changes, including polycyclic aromatic hydrocarbons; heavy metals (nickel, cadmium, chromium and arsenic); carbon monoxide and reactive oxygen species [203]. Well-studied in lung diseases (but never in IBD, to our knowledge), smoking-induced epigenetic modifications seem to be strongly associated with smoking habits, the dose and the duration of smoke exposure [204,205,206,207,208,209]. The methylation of certain genetic loci, post-translational modifications of histones and the level of expressed miR may be reversible after smoking cessation (after 5 years, according to some studies) [93,204,205,206,207,208,209,210]. In contrast to these reversible epigenetic changes, others remain unchanged even after 30 years of smoking cessation, explaining that epigenetic modifications induced by smoking exposition confer long-term risks of adverse health outcomes but could also be transmitted to the next generation [93,204,207,208,209,210,211]. The mechanisms by which tobacco may contribute to inflammation are multiple and involve changes in the enzymes involved in DNA methylation, post-transcriptional histone modifications and ncRNAs [65,93,203,212,213,214].

Regarding smoking-induced DNA methylation, a meta-analysis performed by Joehanes and colleagues highlighted various genome-wide association studies showing that smoking-induced genes differentially methylated are enriched for variants associated with smoking-related diseases, including IBD, CD and UC [210,215,216]. The findings suggest that changes in methylation of the *BCL3*, *FKBP5*, *AHRR* and *GPR15* genes are involved in the mechanism by which smoking increases the risk of CD [217,218].

Concerning smoking-induced histone modifications, smoking also contributes to histone hyperacetylation (H4 histone in active smoking and H3 histones in ex-smokers) by upregulating HATs and downregulating SIRT 1–7 (which belong to the family of class III HDACs) [65,219,220,221,222,223,224,225,226,227]. This imbalance, in favor of histones acetylation, contributes to the increased transcription of proinflammatory genes, mainly controlled by NF-κβ [65,219,221,224,228,229,230,231,232], and the increase of expression of proinflammatory mediators (including IL-1β, TNF-α and IL-6), contributing to chronic inflammation [228,229,233,234]. In a colitis model, Lo and colleagues showed that the reduction of SIRT2 could also be associated with a reduction of the M2-associated anti-inflammatory pathway [229]. The SIRT3 reduction is associated with less activation of the NALP3 inflammasome [235]. Cigarette smoke exposure upregulates the enzyme that catabolizes HMTs, leading to an increase of the H3 and H4 histone residue methylation [226], which may contribute to the proinflammatory cascade [236].

Smoking exposure also alters ncRNAs in a dose-and-time-dependent manner, high doses of and long-lasting exposure being necessary to induce irreversible ncRNA alterations, which may be involved in smoking-related diseases [237,238]. While there are no data on lncRNAs, the impact of smoking on miRs in IBD has been better studied. Interestingly, these IBD-induced epigenetic changes could partly explain why smoking is rather protective in UC, whereas it is an important risk factor in CD. Indeed, nicotine enhances the miR-124 expression, which targets and downregulates IL6R, resulting in a shifting Th1/Th2 balance toward Th1 (in peripheral blood lymphocytes and colon tissues), thereby protecting against Th2-type UC and worsening Th1-type CD [239]. This increase in miR-124 in epithelial cells, lymphocytes and macrophages in response to nicotine also results in the phosphorylation of STAT3, in a decreased production of IL-6 at the transcriptional level, and prevents the conversion of pro-TNF-α to TNF-α, which also explains the protective role of tobacco in the UC [240,241]. Tobacco also induces changes in several miRs that are functionally related to inflammation [65]. Among those highlighted in the IBDs are miR-21, miR-132, miR-195 and miR-223 [65]. MiR-21 (increased in the colon of IBD patients [242]) is known to increase the intestinal epithelial permeability (through an action on the tight junctions) [242,243,244] and plays a crucial role in T-cell differentiation, apoptosis and activation [242,245,246,247] and promotes the production of inflammatory cytokines (including TNF-α, IFN-γ and IL-1β) by immune cells, contributing to tissue inflammation and IBD pathogenesis [248,249,250]. The overexpression of a miR-195 precursor lowered the cellular levels of the Smad7 protein, leading to a decrease in c-Jun and p65 expression, and might contribute to the protective effect of tobacco in UC [251]. Lastly, smoking also downregulates miR-200 [252], known to repress epithelial-to-mesenchymal transition (or EMT), a process involved in intestinal fibrosis [242,252]. Consequently, the decrease of miR-200 in response to smoking could partly explain why smoking IBD patients are more likely to develop intestinal fibrosis (and fibrostenosis) [253,254,255].

### 4.6. Drugs

While the impact of NSAIDs (non-steroidal anti-inflammatory drugs) and oral contraceptives [256,257], whose long-term consumption is known to be associated with IBD, on the epigenome of IBD patients has apparently not yet been investigated, other molecules have a known impact (Table 4). Several treatments used in IBD exert their anti-inflammatory action via epigenetic modifications, such as 5ASA [258,259], anti-TNF [125,127,260,261,262,263,264], exclusive enteral nutrition [265] and mesenchymal stem cells [266,267]. Antibiotics [268,269,270] and probiotics [271,272,273,274,275,276] can also reduce gut inflammation through various epigenetic mechanisms. Finally, a range of Chinese herbs have been shown to have an epigenetically mediated anti-inflammatory action in the gut [277,278,279,280,281,282,283,284,285,286,287].

### 4.7. Vitamin D

Vitamin D is an environmental factor involved in IBD pathogenesis. Its deficiency, which can be both a cause and a consequence of IBD, is associated to an increased risk of disease activity, mucosal inflammation, clinical relapse and a lower quality of life [301]. A vitamin D-deficient diet contributes to IBD through the following epigenetic mechanisms: (1) increase in miR-142-3p expression in intestinal tissues leading to autophagy dysregulation [302]; (2) reduction of the interaction between VDR and HDAC11, an important complex for the maintenance of the epithelial barrier [303], and (3) the upregulation of miR-125b expression and reduction of M1 macrophage polarization to the M2 subtype [304].

### 4.8. Physical Activity

Despite all the known benefits of physical activity (PA) in IBD [16], the way in which it modifies epigenetics has never been studied to date to our knowledge. In other inflammatory diseases, the general view is that regular moderate-intensity physical activity could have an anti-inflammatory effect, while prolonged or high-intensity PA can trigger inflammation, both by leading to epigenetic changes that, in turn, regulate inflammatory responses in peripheral tissues [305,306,307]. These peripheral epigenetic changes appear to be largely induced by the muscle secretome, also known as “myokinome”, which corresponds to all the cytokines or proteins produced by the myocyte in response to muscular contractions [308,309]. These sport-induced epigenetic modulations (including both DNA methylations, histone modifications and miR modulations [309]) seem to vary according to the type of performed exercise (and the frequency, the intensity and the duration) [309]; the individual (in terms of age, gender and body composition) [305,309] and can vary from one tissue to another [310].

Studies suggest that PA is associated with DNA hypermethylation (although these results are not unanimous), contributing to a decreased expression of inflammation-related genes (such as the hypermethylation of IL-17A and IFN-γ promoter regions or TNF gene and the hypomethylation of IL-10) [311]. Exercise can also induce histones acetylation/deacetylation in a body mass index-dependent manner [312]. Finally, physical activity also leads to the release of a range of miRs from the muscle, known to play roles in macrophage polarization, dendritic cell activation, dendritic cell-mediated T-cell activation and the Th1 and Th17 differentiation of T cells, which are all pathophysiological processes involved in IBD [309].

## 5. Limitations to the Analysis of the Exposome Impact on the Epigenome in IBD

The study of the impact of the exposome on the epigenome is difficult because of the limitations of both the exposome study and the epigenome study. Human epidemiological studies are necessary to assess exposome-related epimutation. The first way to study the impact of the exposome is using retrospective case–control epidemiological studies (which compare the life of IBD patients with control cases to identify environmental influences based on surveys), but these studies are subject to a recall bias of past exposure factors. The second way of studying the exposome is a multi-omics approach (via the quantification and detection of external influences in a group of patients compared to a control group by different technologies), but unfortunately, it does not allow the detection of the factors responsible for the crucial pathophysiological changes explaining the occurrence of the pathology, it is very difficult to obtain a cause-and-effect relationship when using this method. A third approach, which counters this, consists of directly studying the supposed factors based on the basis of the pathophysiological hypotheses, but this exposes a selection bias and is not always easy to carry out in humans. Easier to perform on in vivo animal models or in vitro models, it does not reflect what is happening in humans [100,313].

All patients exposed to an environmental factor do not develop epimutation [314,315]. The onset of this and related diseases may depend on the duration, frequency and intensity of exposure to the environmental factor and the period of life during which it occurs [81]. Epigenetic changes can be influenced by age, sex and race but, also, possibly by the underlying host genotype [316,317,318]. Furthermore, the epigenetic changes induced by the exposome may vary according to the cell type and analyzed tissue (peripheral blood mononuclear cells, epithelial cells and biopsies) [316]. The tissue isolation and manipulation may also induce nonspecific epigenetic changes and mask the exposome-related one [100]. Finally, whatever the methodology used, it is always subject to several environmental factors at the same time that may induce concomitant and potentially interactive epigenetic changes, and the impact of an individual factor is not always easy to identify [319].

## 6. Conclusions and Challenge for the Future

**Figure 1:**
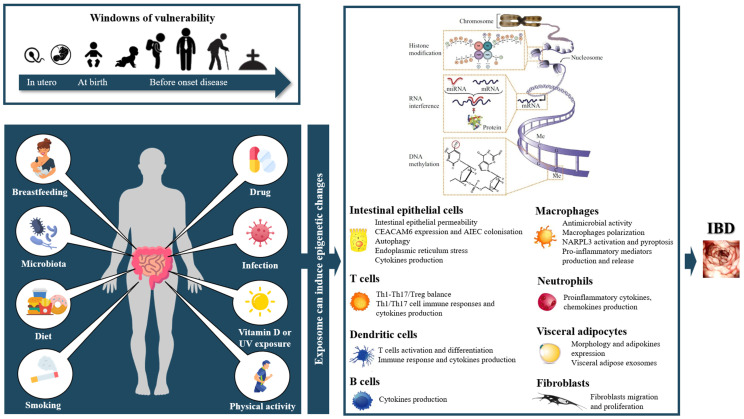
**Summary (adapted from Sawan et al. [323]).** The environmental factors epigenetically influencing the occurrence of intestinal inflammation are breastfeeding, microbiota, diet, smoking habits, drugs, infections, vitamin D and physical activity. Although present at all times, it is mainly during the prenatal period, at birth and just before the onset of the disease that these factors play a key role in triggering the disease. These environmental factors, by inducing DNA methylation, histone modifications and ncRNAs in different cell types, trigger the pathways involved in IBD pathophysiology and contribute to disease initiation.

This review is the first, to our knowledge, to study the impact of the exposome on the epigenome in IBD. Different elements of the exposome such as the maternal lifestyle, microbiota, diet, smoking, infection and vitamin D, as well as different drugs, may induce epigenetic changes related to IBD (Figure 1). Next to these factors, the impact of other environmental factors known to be involved in the pathophysiology of IBD on host epigenetics has not yet been studied. The influence of physical activity [16], appendicectomy in UC [16], processed and fast food or dietary [320,321] or psychologic stress/anxiety/depression [19], NSAIDs [256], oral contraception [257] or infections [20], as well as other factors potentially involved, deserve to be investigated. Regarding the identified environmental factors, how environmental exposure (in terms of the duration, frequency and timing at which it occurs) induces an epimutation, and whether this involves an exposome–microbiome–epigenome axis and becomes a critical factor for IBD development is largely unknown and remains to be further investigated. Finally, the exposome could be a tool to predict relapses [322]. The development of electronic technologies to continuously record a patient’s exposome could allow disease-modifying exposures to be detected and acted on early to prevent relapse or disease progression [322].

## Figures and Tables

**Table 2:** **Impact of the gut microbiota-derived metabolites on the epigenome in intestinal inflammation**. CD, Crohn’s disease; CEBPB, CCAAT/enhancer binding protein; DNA, deoxyribonucleic acid; DSS, dextran sulfate sodium; HDAC, histone deacetylases; IBD, inflammatory bowel disease; IECs, intestinal epithelial cells; IFN, interferon; IL, interleukin; lncRNAs, long non-coding RNAs; LPS, lipopolysaccharide; MCP-1, Monocyte chemoattractant protein-1; miR, micro-RNA; NF-κB, nuclear factor-kappa B; NOD2, nucleotide-binding oligomerization domain 2; SCFAs, short-chain fatty acids; STAT, signal transducer and activator of transcription; TNF, tumor necrosis factor; UC, ulcerative colitis.

Metabolite	Activity	Epigenetic Mechanism	Tissue/Cells	Mechanism	Model	Author
**SCFAs**						
SCFAs	Anti-inflammatory activity	HDACs inhibition	T cells	Inhibits HDACs in T cells and increases the acetylation of p70 S6 kinase and phosphorylation rS6, regulating the mTOR pathway required for generation of Th17 (T helper type 17), Th1, and IL-10(+) T cells	C57BL/6 mice; CD4+ T cells isolated from the spleen and lymph nodes	Park J, et al. (2015) [154]
SCFAs	Anti-inflammatory activity	HDACs inhibition	B cells	Upregulates regulatory B cells capable of producing IL-10 in a manner dependent on their HDAC inhibitory activity	DSS-induced colitis in mice	Zou F, et al. (2021) [155]
SCFAs	Anti-inflammatory activity	miR-145	IECs	Decreases the CEBPB expression, which could bind to the miR-145 promoter to inhibit its expression, thereby promoting the expression of DUSP6 (dual-specificity phosphatase 6) and thus prevents the development of intestinal inflammation	LPS-treated intestinal epithelial cells	Liu Q, et al. (2022) [156]
**Butyrate**						
Butyrate	Anti-inflammatory activity	Histone acetylation	IECs	Butyrate, by inducing an increase in histone acetylation in the NOD2 promoter region, induces NOD2 upregulation, and impact the defence mechanism against the bacterial membrane component peptidoglycan by inducing IL-8 and GRO-alpha secretion	Caco-2 cell line	Leung CH, et al. (2009) [147]
Butyrate	Anti-inflammatory activity	HDAC inhibition	Dendritic cells	Butyrate has a role of HDACi on the epigenetic modification of gene expression, inhibits IL-12 and upregulates subunit IL-23p19	DSS-induced colitis in mice	Berndt BE, et al. (2012) [153]
Butyrate	Anti-inflammatory activity	HDAC1 inhibition	T cells	Butyrate inhibits HDAC1 activity to induce Fas promoter hyperacetylation and Fas upregulation in T cells and promote Fas-mediated apoptosis of T cells to eliminate the source of inflammation	BALB/c mice	Zimmerman MA, et al. (2012) [157]
Butyrate	Anti-inflammatory activity	HDAC inhibition	IECs	Butyrate may contribute to the restoration of the tight junction barrier in IBD by affecting the expression of claudin-2, occludin, cingulin, and zonula occludens proteins (ZO-1, ZO-2) via inhibition of histone deacetylase	DSS-induced colitis in mice	Plöger S, et al. (2012) [146]
Butyrate	Anti-inflammatory activity	Histone H3 acetylation	T cells	Butyrate enhances histone H3 acetylation in the promoter and conserves non-coding sequence regions of the *Foxp3* locus, regulating the differentiation of Treg cells, ameliorating colitis	Germ-free and CRB-associated mice; OT-II (Ly5.2) transgenic CD4+ T cells	Furusawa Y, et al. (2013) [144]
Butyrate	Anti-inflammatory activity	HDAC inhibition	Macrophages	Butyrate reduces de production of proinflammatory mediators by macrophages including nitric oxide, IL-6, and IL-12, but did not affect levels of TNF-α or MCP-1	DSS-induced colitis in mice	Chang PV, et al. (2014) [149]
Butyrate	Anti-inflammatory activity	H3K9 acetylation	Macrophages	Butyrate activates STAT6-mediated transcription through H3K9 acetylation driving M2 macrophage polarization	DSS-induced colitis in mice	Ji J, et al. (2016) [150]
Butyrate	Anti-inflammatory activity	Histone H3 acetylation	Macrophages	Oral supplementation with butyrate attenuates experimental murine colitis by blocking NF-κB signaling and reverses histone acetylation	DSS-induced colitis in mice, IL-10^−/−^ mice and RAW264.7 cells	Lee C, et al. (2017) [158]
Butyrate	Anti or proinflammatory activity depending on its concentration and immunological milieu	HDACs inhibition	T cells	Lower butyrate concentrations facilitates differentiation of Tregs in vitro and in vivo under steady-state conditions. In contrast, higher concentrations of butyrate induces expression of the transcription factor T-bet in all investigated T cell subsets resulting in IFN-γ-producing Tregs or conventional T cells. This effect was mediated by the inhibition of histone deacetylase activity	DSS-induced colitis in mice; CD4+ T cells	Kespohl M, et al. (2017) [159]
Butyrate	Anti-inflammatory activity	HDAC3 inhibition	MonocyteMacrophage	Butyrate induces the monocyte to macrophage differentiation and promotes its antimicrobial activity and restricts bacterial translocation, through HDAC3 inhibition	Human monocytes isolated from leukocyte cones of healthy blood donors	Schulthess J, et al. (2019) [148]
Butyrate	Anti-inflammatory activity	HDAC inhibition	T cells	Butyrate promotes Th1 cell development by promoting IFN-γ and T-bet expression and inhibits Th17 cell development by suppressing IL-17, Rorα, and Rorγt expression and upregulate IL-10 production in Th1 and Th17	CBir1 transgenic T cells; Rag1^−/−^ mice	Chen L, et al. (2019) [151]
Butyrate	Anti-inflammatory activity	Increase of histone acetylation	IECs	Butyrate induces HSF2 (Heat-shock transcription factor 2) expression epigenetically via increasing histone acetylation levels at the promoter region, enhancing autophagy in IECs	UC (*n* = 50) and healthy (*n* = 30) patients; DSS-induced colitis in mice; HT-29 cells	Zhang F, et al. (2020) [160]
Butyrate	Anti-inflammatory activity	HDAC inhibition	IECs	Butyrate induces SYNPO (Synaptopodin) in epithelial cell lines through mechanisms possibly involving histone deacetylase inhibition. SYNPO contributes by intestinal homeostasis by controlling intestinal permeability	Epithelial cell lines; DSS-induced colitis in mice	Wang RX, et al. (2020) [161]
Butyrate	Anti-inflammatory activity	HDAC inhibition	Neutrophils	Butyrate significantly inhibits IBD neutrophils to produce proinflammatory cytokines, chemokines, and calprotectins through HDAC inhibition	Peripheral neutrophils isolated from IBD patients and healthy donors; DSS-induced colitis in mice	Li G, et al. (2021) [152]
Propionate	Anti-inflammatory activity	HDAC1 inhibition	IECs	Propionate promotes intestinal epithelial cell migration by enhancing cell spreading and polarization, a function dependant of the inhibition of class I HDAC	Mouse small intestinal epithelial cells (MSIE) and human Caco-2 cells; DSS-induced colitis in mice	Bilotta AJ, et al. (2021) [142]
Caprylic acid (C8) and nonanoic acid (C9) (medium chain fatty acids)	Anti-inflammatory activity	Acetylation of histone 3 lysine 9 (H3K9)	IECs	Reduces bacterial translocation, enhances antibacterial activity, and attenuates the activity of the classical histone deacetylase pathway to facilitate the acetylation of histone 3 lysine 9 (H3K9) at the promoters pBD-1 and pBD-2, remarkably increases the secretion of porcine β-defensins 1 (pBD-1) and pBD-2	Porcine jejunal epithelial cell line-J2	Wang J, et al. (2018) [162]

**Table 4:** **Impact of drugs on epigenome in intestinal inflammation**. 5-ASA, 5-aminosalicylic acid; CD, Crohn’s disease; circRNA, circular RNA; CpG, CpG, cytosine–phosphate–guanine; DNA, deoxyribonucleic acid; DNMT, DNA methyltransferase; DSS, dextran sulfate sodium; EEN, Exclusive enteral nutrition; EMT, epithelial-to-mesenchymal-transition; EV, extracellular vesicle; HDAC, histone deacetylases; HPM, Herb-partitioned moxibustion; IBD, inflammatory bowel disease; IEC, intestinal epithelial cell; IFN, interferon; IL, interleukin; lncRNA, long non-coding RNA; LPS, lipopolysaccharide; miR, micro-RNA; MSC, mesenchymal stem cell; PPAR-γ, peroxisome proliferator-activated receptor γ; RNA, ribonucleic acid; SCFAs, short-chain fatty acids; SNIP1, Smad Nuclear Interacting Protein 1; STAT, signal transducer and activator of transcription; TLR, toll-like receptor; TNF, tumor necrosis factor; TNBS, 2,4,6-Trinitrobenzene sulfonic acid; UC, ulcerative colitis; VEGF, Vascular Endothelial Growth Factor; ZO, zonula occludens.

Drug	Activity	Epigenetic Mechanism	Tissue/Cells	Mechanism	Model	Author
**IBD medication**						
Mesalamine	Anti-inflammatory activity	miR-206	IECs and colonic tissues	Long-term treatment donw-regulates miR-206 which confer a protective effect in inducing and maintaining histologic remission	HT29 colon cells; UC patients (*n* = 10)	Minacapelli CD, et al. (2019) [258]
5-ASA	Anti-inflammatory activity	miR-125b, miR-150, miR-155, miR-346 and miR-506	IECs	5-ASA suppressed the levels of miR-125b, miR-150, miR-155, miR-346 and miR-506 in IECs and inhibition of these miR were associated with significant inductions of their target genes such as vitamin D receptor (VDR), suppressor of cytokine signaling (SOCS1), Forkhead box O (FOXO3a) and DNA methyltransferase 1 (DNMT1)	Caco-2 cells	Adamowicz M, et al. (2021) [259]
Infliximab	Anti-inflammatory activity	miR-10a	DCs	Anti-TNF mAb treatment significantly promote miR-10a expression, whereas it markedly inhibited NOD2 and IL-12/IL-23p40 in the inflamed mucosa	Human monocyte-derived dendritic cells (DC); IBD patients	Wu W, et al. (2015) [127]
Infliximab	Anti-inflammatory activity	miR-301a	T cells	Decreases miR-301a expression in IBD CD4+ T cells by decreasing Th17 cell differentiation through upregulation of SNIP1	Peripheral blood mononuclear cells (PBMC); inflamed mucosa of patients with IBD	He C, et al. (2016) [260]
Infliximab	Anti-inflammatory activity	lnc-ITSN1-2	T cells	Lnc-ITSN1-2 promotes IBD CD4+ T cell activation, proliferation, and Th1/Th17 cell differentiation by serving as a competing endogenous RNA for IL-23R via sponging miR-125a	Intestinal mucosa from IBD patients (*n* = 6) and healthy controls (*n* = 6)	Nie J, et al. (2020) [261]
Infliximab	Anti-inflammatory activity	miR-30 family	IECs	Decreases circRNA_103765 expression, which act as a molecular sponge to adsorb the miR-30 family and impair the negative regulation of Delta-like ligand 4 (DLL4) and protect human IECs from TNF-α-induced apoptosis	IBD patients; PBMCs	Ye Y, et al. (2021) [262]
Infliximab	Anti-inflammatory activity	miR-146a and miR-146b	Serum and intestinal mucosae	Decreases miR-146a and miR-146b levels in serum. miR-146a probably promotes colitis through TLR4/MyD88/NF-κB signaling pathway	Serum of 19 IBD patients	Batra SK, et al. (2020) [263]
Infliximab (IFX) therapy and longer-term steroids (weeks)	Anti-inflammatory activity	miR-320a		Decreases miR-320a serum level. miR-320a could play a role in sensitization of the quiescent mucosa to environmental factors	Serum of 19 IBD patients	
Anti-TNF and glucocorticoids	Anti-inflammatory activity	let-7c		let-7c serum level decreases, thus reduces M2 macrophage polarization (anti-inflammatory) and promote M1 (proinflammatory) polarization	Serum of 19 IBD patients	
Anti-TNF	Anti-inflammatory activity	miR-10a	DCs	Blockade TNF with anti-TNF mAb markedly enhances miR10a expression in the intestinal mucosa. miR-10a could block intestinal inflammation and reduce the differentiation Th1 and Th17	C57BL/6 (B6) mice	Xue X, et al. (2011) [125]
Anti-TNF	Anti-inflammatory activity	miR-378a-3p, miR-378c	Colonic mucosae	Increases levels of miR-378a-3p and miR-378c. Over-expression of miR-378a-3p decreased the levels of an IL-33 target sequence β-gal-reporter gene	Active UC patients (*n* = 24); inactive UC (*n* = 10); controls (*n* = 6); HEK293 cells	Dubois-Camacho K, et al. (2019) [264]
Enemas containing short chain fatty acids (SCFA) such as butyrate, propionate, and acetate	Anti-inflammatory activity	Histone acetylation	IECs	SCFAs increase histone acetylation states and inhibit the production of proinflammatory substances, such as IL-8, by the intestinal epithelium	Caco-2 cells	Huang N, et al. (1997) [288]
N-(1-carbamoyl-2-phenylethyl) butyramide (FBA), a butyrate-releasing derivative	Anti-inflammatory activity	Histone deacetylase-9 and H3 histone acetylation	Colonic mucosae	FBA, similar to its parental compound sodium butyrate, inhibited histone deacetylase-9 and restored H3 histone acetylation, exerting an anti-inflammatory effect through NF-κB inhibition and the upregulation of PPARγ	DSS-induced colitis in mice	Simeoli R, et al. (2017) [289]
Exclusive enteral nutrition (EEN)	Anti-inflammatory activity	hsa-miR-192-5p, hsa-miR-423-3p, hsa-miR-99a-5p, hsa-miR-124-3p, hsa-miR-301a-5p, hsa-miR-495-5p, and hsa-let-7b-5p	Intestinal mucosae	EEN induces mucosal miRNAs expression profile (altered expressions of hsa-miR-192-5p, hsa-miR-423-3p, hsa-miR-99a-5p, hsa-miR-124-3p, hsa-miR-301a-5p, hsa-miR-495-5p, and hsa-let-7b-5p) after EEN therapy was significantly changed compared with inflamed mucosa before treatment	CD patients (*n* = 30)	Guo Z, et al. (2016) [265]
ABX464	Anti-inflammatory activity	miR-124	Immune cells	Upregulates miR-124 in human immune cells, which is a negative regulator of inflammation and was shown to target RNAs, such as STAT and TLR		Tazi J, et al. (2021) [290]
MSCs	Anti-inflammatory activity	miR-181a	IECs	MSC-derived exosomal miR-181a could alleviate colitis by promoting intestinal barrier function decreased (increasing level of Claudin-1, ZO-1, and IκB)	DSS-induced colitis in mice and induced human colonic epithelial cell (HCOEPIC)	Gu L, et al. (2021) [266]
MSCs	Anti-inflammatory activity	H3K27me3	T cells	Extracellular vesicles from MSCs could inhibit the differentiation of Th17 cells by regulating H3K27me3	TNBS-induced colitis in mice	Chen Q, et al. (2020) [291]
IFN-γ pretreated bone marrow mesenchymal stem cells	Anti-inflammatory activity	miR-125a and miR-125b	T cells	Increases the level of miR-125a and miR-125b of exosomes, which directly targeted on Stat3, to repress Th17 cell differentiation	DSS-induced colitis in mice	Yang R, et al. (2020) [267]
Vascular endothelial growth factor-C-treated adipose-derived stem cells (ADSCs)	Anti-inflammatory activity	miR-132	Lymphatic endothelial cells	VEGF-C-treated ADSCs have a higher level of miR-132, which promotes lymphangiogenic response by directly targeting Smad-7 and regulating TGF-β/Smad signaling	Lymphatic endothelial cells (LECs)	Wang X, et al. (2018) [292]
**Supplementation**						
Iron	Proinflammatory activity	TET1 induction; NRF2, NQ01, GPX2 demethylation	IECs and intestinal mucosae	Chronic iron exposure leads to induction of TET1 expression leading to demethylation of NRF2 (nuclear factor erythroid 2-related factor 2) pathway targets (including NAD(P)H Quinone Dehydrogenase 1 (NQO1) and Glutathione peroxidase 2 (GPX2). NQO1 and GPX2 hypomethylation led to increased gene and protein expression, and could be a route by which cells overcome persistent and chronic oxidative stress	Caco-2 cells and wild-type C57BL/6 mice	Horniblow RD, et al. (2022) [293]
**Antibiotics**						
Isotretinoin	Anti-inflammatory activity	miR	T cells	3 miR overexpressed in naive T-cells and potentially downregulate 777 miR targets (cytoskeleton remodelling and the c-Jun N terminal kinase (JNK) signaling pathway)	Balb/c mice	Becker E, et al. (2016) [268]
Metronidazole	Anti-inflammatory activity	miR		5 miR were significantly lower in naive T-cells resulting in the prediction of 340 potentially upregulated miR targets associated with IL-2 activation and signaling, cytoskeleton remodelling and epithelial-to-mesenchymal-transition (EMT).		
Doxycycline	Anti-inflammatory activity	miR-144-3p		Overexpression of miR-144-3p that resulted in the prediction of 493 potentially downregulated miR targets involved in protein kinase A (PKA), protein kinase B and nuclear factor of activated T-cells (NFAT) signaling pathways		
Tetracyclines	Anti-inflammatory activity	miR-150, miR-155, miR-375 and miR-146	Colonic tissues	Reduce miR-150 and miR-155 expression, upregulate miR-375 and miR-142	DSS-induced colitis in mice and bone marrow-derived macrophages	Garrido-Mesa J, et al. (2018) [269]
Antibiotics treatment	Anti-inflammatory activity	DNA demethylation	IECs	Suppresses aberrant DNA methylation of three marker CpG islands (Cbln4, Fosb, and Msx1) induced by chronic inflammation	AOM/DSS-induced colitis in mice	Hattori N, et al. (2019) [270]
**Probiotics**						
Probiotic bacterium *Escherichia coli* Nissle 1917 (EcN)	Anti-inflammatory activity	miR-203, miR-483-3p, miR-595	IECs	Increases miR-203, miR-483-3p, miR-595 targeting tight junction (TJ) proteins; these miRNAs are involved in the regulation of barrier function by modulating the expression of regulatory and structural components of tight junctional complexes.	T84 cells	Veltman K, et al. (2012) [271]
Bifidobacterium longum	Anti-inflammatory activity	DNA demethylation	Peripheral blood mononuclear cells	B. Longum treatment significantly demethylates several CpG sites in Foxp3 promoter	TNBS-induced colitis in rat; spleen peripheral blood mononuclear cells (PBMC) cells was extracted	Zhang M, et al. (2017) [272]
Lactobacillus fermentum and Lactobacillus salivarius	Anti-inflammatory activity	miR-155, miR-223, miR-150 and miR-143	Colonic tissues	They increase the expression of miR-155 and miR-223, and miR-150 and miR-143 for L. fermentum, involved in the immune response (restoration of Treg cell population and the Th1/Th2 cytokine balance) and in the intestinal barrier function	C57BL/6J mice	Rodríguez-Nogales A, et al. (2017) [273]
Saccharomyces boulardii	Anti-inflammatory activity	miR-155 and miR-223; miR-143 and miR-375	Colonic tissuess	Increasing the expression of miR-155 and miR-223, whereas decreasing the expression miR-143 and miR-375	DSS-induced colitis in mice	Rodríguez-Nogales A, et al. (2018) [274]
Bifidobacterium bifidum ATCC 29521	Anti-inflammatory activity	miR-150, miR-155, miR-223	Colonic mucosae	Restorates miR-150, miR-155, miR-223, upregulates anti-inflammatory cytokines (IL-10, PPARγ, IL-6), tight junction proteins (such as ZO-1, MUC-2, Claudin-3, and E Cadherin-1) and downregulates inflammatory genes (TNF-α, IL-1β)	DSS-induced colitis in mice	Din AU, et al. (2020) [275]
Lactobacillus casei LH23 probiotic	Anti-inflammatory activity	Histone H3K9 acetylation	Colonic tissues	Modulates the immune response and ameliorates colitis via suppressing JNK/p-38 signal pathways and enhancing histone H3K9 acetylation	DSS-induced colitis in mice; LPS-induced RAW264.7 cells	Liu M, et al. (2020) [294]
Lactic Acid-Producing Probiotic *Saccharomyces cerevisiae*	Anti-inflammatory activity	Histone H3K9 acetylation and histone H3K18 lactylation	Macrophages	Promotes histone H3K9 acetylation and histone H3K18 lactylation and attenuates intestinal inflammation via suppressing macrophage pyroptosis	DSS-induced colitis in mice	Sun S, et al. (2021) [276]
**Other medication**						
Telmisartan (angiotensin II type 1 receptor blocker and a peroxisome proliferator-activated receptor-receptor-γ agonist)	Anti-inflammatory activity	miR-155	Mesenteric adipocytes	Restorates the mesenteric adipose tissue adipocyte morphology and the expression of adipokines by suppressing the neurotensin/miR-155 pathway	IL-10^(−)/(−)^ mice; cultured mesenteric adipose tissue from Crohn’s disease patients	Li Y, et al. (2015) [295]
Melatonine	Anti-inflammatory activity	Prevent DNA methylation	IECs	Prevents DNA demethylation, reduces NF-κB activation, decreases the levels of inflammatory mediators (including IL-6, IL-8, COX-2, and NO), and reduces increase in paracellular permeability, attenuating the inflammatory response	Caco-2 cells	Mannino G, et al. (2019) [296]
Morphine	Proinflammatory activity	Let7c-5p	Macrophages, DCs	Opioid treatment can disrupt gut immune homeostasis by inhibiting packaging of miR into EVs secreted by intestinal crypt cells (with a decreased amount of Let7c-5p)	C57BL/6J mice; organoid culture	Zhang Y, et al. (2021) [297]
Artesunate	Anti-inflammatory activity	miR-155	Macrophages	Inhibits the expression of miR-155 to inhibit the NF-κB pathway	LPS-induced RAW264.7 cells; BALB/c mice model	Yang ZB, et al. (2021) [298]
Valproic acid treatment	Anti-inflammatory activity	HDAC inhibition	Intestinal tissue	Inhibits HDAC activity and increases H3K27ac levels and reduced expression of IL6, IL10, IL1B, and IL23	DSS-induced colitis in mice	Felice C, et al. (2021) [299]
Tetrandrine	Anti-inflammatory activity	miR-429	IECs	Tetrandrine can attenuate the intestinal epithelial barrier defects in colitis through promoting occludin expression via the AhR/miR-429 pathway	DSS-induced colitis in mice	Chu Y, et al. (2021) [300]
**Chinese medicine**						
Sinomenine, a pure alkaloid isolated in Chinese medicine	Anti-inflammatory activity	miR-155	Colonic tissues	Downregulates the levels of miR-155 and several related inflammatory cytokines	TNBS-induced colitis in mice	Yu Q, et al. (2013) [277]
Tripterygium wilfordii Hook F (TWHF)	Anti-inflammatory activity	miR-155	Ileocolonic anastomosis	Triptolide could suppress miR-155/SHIP-1 signaling pathway and attenuated expression of inflammatory cytokines after ileocaecal resection	IL-10^(−/−)^ mice	Wu R, et al. (2013) [278]
Herb-partitioned moxibustion (HPM)	Anti-inflammatory activity	miR-147 and miR-205	Colonic tissues	Upregulates the expression of miR-147 and miR-205 and then further regulate some of their target genes, thereby indirectly inhibiting the inflammatory signal pathways mediated by TLR, NF-κB, and so forth and decreasing the production of downstream inflammatory cytokines such as TNF-α and IL-1β, so as to alleviate intestinal inflammation in CD	Experimental CD rat models	Wei K, et al. (2015) [280]
Salvianolic acid B (Sal B) is isolated from the traditional Chinese medical herb Salvia miltiorrhiza	Anti-inflammatory activity	miR-1	IECs	Sal B restores barrier function by miR-1 activation and subsequent myosin light chain kinase (MLCK) inactivation	TNBS-induced rat colitis model	Xiong Y, et al. (2016) [281]
Herb-partitioned moxibustion (HPM)	Anti-inflammatory activity	miR-184 and miR-490-5p	Colonic tissue	HPM regulates miR-184 and miR-490-5p expression, act on the transcription of their target genes to regulate inflammatory signaling pathways, and attenuate inflammation and tissue injury in the colons of rats with DSS-induced UC	DSS-induced colitis in mice	Huang Y, et al. (2017) [282]
Triptolide (TPL)	Anti-inflammatory activity	miR-16-1	Ileocolonic anastomosis	TPL reduces miR-16-1 levels aggravating anastomotic inflammation and fibrosis	IL-10^−/−^ mice	Hou HW, et al. (2017) [283]
Norisoboldine (NOR), a natural aryl hydrocarbon receptor (AhR)	Anti-inflammatory activity	H3K9me3 modification	T cells	NOR promoted Treg differentiation and then alleviated the development of colitis by regulating AhR (aryl hydrocarbon receptor)/glycolysis axis and decreases NAD+ and SIRT1 (sirtuin 1), facilitates the ubiquitin-proteasomal degradation of SUV39H1, which is a major member of histone KMTs and catalyses the H3K9me3 modification, which is associated with transcription repression of Foxp3		Lv Q, et al. (2018) [284]
Triptolide (TPL), the most potent bioactive substance in TWHF (*Tripterygium wilfordii* Hook F) extract	Anti-inflammatory activity	miR-16-1	Fibroblasts	Inhibits migration and proliferation of fibroblasts from ileocolonic anastomosis of CD patients via regulating the miR-16-1/HSP70 pathway	Fibroblasts from strictured anastomosis tissue (SAT) samples and matched anastomosis-adjacent normal tissue (NT) samples of CD patients (*n* = 10)	Chen M, et al. (2019) [285]
Polysaccharide RAMPtp from Atractylodis macrocephalae Koidz	Anti-inflammatory activity	lncRNA ITSN1-OT1	IECs	Induces lncRNA ITSN1-OT1, which blocks the nuclear import of phosphorylated STAT2 and prevents the decrease of expression and structural destroy of tight junction proteins	DSS-induced colitis in mice	Zong X, et al. (2021) [286]
Dendrobium officinale polysaccharide (DOP)	Anti-inflammatory activity	miR-433-3p	IECs, macrophages	DOP interfered with the secretion of small extracellular vesicles (DIEs) by IEC, with increased miR-433-3p expression. When delivered to macrophages, miR-433-3p targeted the MAPK8 gene, leading to inhibition of the MAPK signaling pathway and reduced production of inflammatory cytokines	IECs, macrophages	Liu H, et al. (2021) [287]
Huangqin-Tang decoction (HQT)	Anti-inflammatory activity	miR-185-3p	IECs	HQT could upregulate miR-185-3p, thereby affecting the myosin light chain kinase (MLCK)/myosin light chain phosphorylation (p-MLC) pathway and leading to increased expression of occludin protein, which ultimately protected the intestinal epithelial barrier function	Balb/c mice	Changlin Z, et al. (2021) [279]

## Abbreviations

5hmC, 5-hydroxymethyl cytosine; ADAM17, a disintegrin and metalloprotease17; AIEC, Adherent-invasive Escherichia coli; CD, Crohn’s disease; CEACAM6, CEA Cell Adhesion Molecule 6; CpG, cytosine-phosphate-guanine; DNA, deoxyribonucleic acid; DNMT, DNA methyltransferase; EMT, epithelial-to-mesenchymal transition; HAT, histone acetyltransferase; HDAC, histone deacetylases; HDM, histone demethylases; HMT, histone methyltransferase; HRE, HIF-1-responsive elements; IBD, inflammatory bowel disease; IEC, intestinal epithelial cell; IL, interleukin; Jnk, c-Jun N-terminal kinase; LPS, lipopolysaccharide; lncRNA, long non-coding RNA; MAP, Mycobacterium avium subspecies paratuberculosis; MeSH, Medical Subject Heading; miR, micro-RNA; mRNA, messenger RNAs; ncRNA, non-coding RNA; NF-κB, nuclear factor-kappa B; NLRP3, NOD-like receptor family pyrin domain containing 3; NOD2, nucleotide-binding oligomerization domain 2; NSAID, non-steroidal anti-inflammatory drug; PPAR-γ, peroxisome proliferator-activated receptor γ; RNA, ribonucleic acid; SAM, S-adenosyl-L-methionine; SCFA, short-chain fatty acid; SIRT, sirtuin; STAT, signal transducer and activator of transcription; TET, ten-eleven translocation enzymes; TNF, tumor necrosis factor; UC, ulcerative colitis.
